# Local Residents’ Perceptions for Ecosystem Services: A Case Study of Fenghe River Watershed

**DOI:** 10.3390/ijerph16193602

**Published:** 2019-09-26

**Authors:** Hongjuan Zhang, Qian Pang, Huan Long, Haochen Zhu, Xin Gao, Xiuqing Li, Xiaohui Jiang, Kang Liu

**Affiliations:** 1 College of Urban and Environmental Sciences, Northwest University, Xi’an 710127, China; 201710179@stumail.nwu.edu.cn (H.Z.); 201731726@stumail.nwu.edu.cn (Q.P.); 201731771@stumail.nwu.edu.cn (H.L.); heracles_z@stumail.nwu.edu.cn (H.Z.); lixiuqingscsfdx@163.com (X.L.); xhjiang@nwu.edu.cn (X.J.); 2 Shaanxi Key Laboratory of Earth Surface System and Environmental Carrying Capacity, Xi’an 710127, China; 3 Business School, Hohai University, Nanjing 211100, China; gxtz1987@hhu.edu.cn

**Keywords:** ecosystem services, social assessment approach, social preference, willingness to pay, preference heterogeneity

## Abstract

To make environmental management decisions more executive and targeted, it is essential for decision-making to include local residents’ perceptions and preferences for ecosystem services (ES) and biodiversity (BD). This study conducted a questionnaire survey with 386 local residents to explore social perceptions for ES and BD in the Fenghe River watershed. ES contain food from agriculture (AGR), food from livestock (LIV), fresh water (FW), air purification (AP), water purification (WP), water retention (WR), soil retention (SR), aesthetics (AES), recreation (RE), and spirit (SP) in this study. Ranking and Likert scales are combined to identify residents’ preferences for ES and BD. The hypothetical market method is used to identify the willingness to pay for BD and ES. Independent sample T-test, one-way ANOVA, and Spearman correlation are used to analyze preference heterogeneity. The results show that: (1) residents prefer WP, AP, AGR, and FW; (2) 51.3% of respondents are willing to pay a fee for improving ES while 48.7% of respondents are unwilling to pay; (3) residents’ personal and residential factors affect preference heterogeneity. Last, we put forward three management suggestions on controlling environmental pollution, improving residents’ awareness of ES, and establishing a multi-channel fund based on government financial resources for improving ES in the Fenghe River watershed. Integrating stakeholders’ perceptions for ES into decision-making can promote the sustainable development in Fenghe River watershed.

## 1. Introduction

Ecosystem services (ES) are tangible and intangible products or services that humans obtain from ecosystems [1,2]. However, with population growth and economic development, the earth’s surface land cover has undergone tremendous changes. Agricultural landscapes and human settlements account for 75% of the earth’s ice-free surface [3]. Changes in land cover patterns have changed ecosystem functions and processes and have a significant impact on ES [4,5]. Although the provisioning services of ES have increased, the regulating services and biodiversity (BD) have degraded [6,7,8]. Countries or regions usually solve the problem of ecological damage by establishing nature reserves [9], and more than 12% of the earth’s land surface is designated as protected areas [10].

Consequently, the protection of natural resources has become a global problem. However, the actual protection of natural resources is not as effective as we expected [11]. Usually, the protection of natural resources involves different stakeholders, and these stakeholders have different attitudes and perceptions about the protection of natural resources [12]. According to the existing literature [13], stakeholders’ different perceptions of the environment could affect their different behaviors towards natural environmental protection. When stakeholders have a high awareness of conservation, they may actively protect natural resources; otherwise, they may be passive. However, in the actual environmental protection policy-making process, policy makers usually do not consider the views of other stakeholders when formulating natural resource protection policies, which leads to poor policy implementation [14,15]. To solve more and more ecological problems, stakeholders are encouraged to participate in the decision-making process [16,17,18], which is more conducive to the smooth implementation of policies than top-down policy approaches [19]. 

Recently, many researchers have studied stakeholders’ perceptions for ES [20,21,22,23,24]. The stakeholders’ perceptions for ES were defined as the understandings and the views of stakeholders about the concept of ES. The aims of these studies are mainly divided into three aspects: (1) Using the perceptions of stakeholders to assess the non-use value of ES. For example, Sherrouse et al. [23] used stakeholders’ perceptions to evaluate the value index and spatial distribution of various social values of forest park; (2) Using stakeholders’ perceptions for land use or landscape to assess human demand for ES. For example, Burkhard et al. [25] used a 0–5 scale matrix to quantify human demand for ES. Castillo-Eguskitza et al. [26] used a contingent valuation method to quantify the human demand for ES; (3) Quantifying the willingness of stakeholders to pay for ES. For example, Thompson et al. [17] studied stakeholders’ preferences for mangrove ES and found that respondents’ willingness to pay for local services was higher than global services.

However, people’s perceptions for ES has a certain regional nature, which is closely related to local geographical conditions, cultural characteristics, life experiences, moral beliefs, economic development levels, and so on [27]. Therefore, case studies are important for identifying regional differences and finding regional commonalities [28]. Human society and local ecological environment constitute a social-ecosystem. Understanding the complex relationship between society and ecosystem scientifically and establishing a bottom-up knowledge system can help to support decision-making and reduce decision-making errors. On the basis of the existing literatures, there are differences in the perceptions of ES among stakeholders in different regions. Some authors found that rural residents have a higher awareness of provisioning services than urban residents. They believed that there was a disconnect between human environment and human welfare of provisioning services for urban residents [29]. Some authors argued that rural residents were more aware of regulating services and cultural services than provisioning services because they had important ecological knowledge of ES provided by the environment [30]. However, some authors argued that two hypotheses had been demonstrated in their studies [31]. These researchers identified that different regional stakeholders may have had different understandings of ES, which fully proved that the social ecosystem was a complex system, and regional stakeholders’ understandings of ES could not be simply replicated [32]. 

Sustainable supply of ES was essential for regional sustainable management objectives [33]. Local-scale assessment of ES is urgent and critical for regional decision-making, especially for developing countries [33]. At present, ecological methods and economic methods to evaluate ES have been developed and applied [34,35]. However, there is still a lack of research on ES using social methods [36,37]. Compared with ecological and economic methods, social methods can better reflect complex social-ecosystem [38]. To ensure the sustainable provision of ES on which human beings depend, stakeholders’ perceptions of ES need to be integrated into regional sustainable strategies and management [39,40]. At present, social methods used to study stakeholders’ perceptions for ES have begun to be implemented [41,42]. However, there is still a gap in the study of whether residential factors have an impact on stakeholders’ perceptions for ES.

We selected a watershed, Fenghe River watershed, to study stakeholders’ perceptions for ES and BD. There is an environmental gradient in the Fenghe River watershed, which transits from mountain areas with high forest coverage to plain areas. The vegetation coverage in mountainous areas is higher, and the artificial landscape in plain areas is obvious [43]. Therefore, on the basis of factors such as social population [31,44], we have included more residential factors (distance from the river, distance from the Xi’an City center, habitation, and residential time) besides the residential area to study the residents’ perceptions for ES. The residential area is divided into the upstream area, midstream area, and downstream area, and habitation is divided into urban and rural areas. Perceptions such as the levels of residents’ awareness of ES, and perceived location and the demographic characteristics of residents influence their preferences for ES [21,22]. In this study, we define “perceptions” as the importance of ES perceived by local residents and their willingness to pay for improving ES, and we also call the importance of ES perceived by residents as their preferences for ES. 

The main purpose of this study is to assess the ES preferences perceived by local residents (residential time ≥ 1 year) and their willingness to pay for improving ES in the Fenghe River watershed. Our objectives are: (1) to assess residents’ preference for ES; (2) to identify residents’ willingness to pay for improving ES; (3) to identify factors affecting preferences and preference heterogeneity; (4) to propose some suggests for sustainable ES management in the Fenghe River watershed.

## 2. Materials and Methods 

### 2.1. Study Area

**Figure 1:**
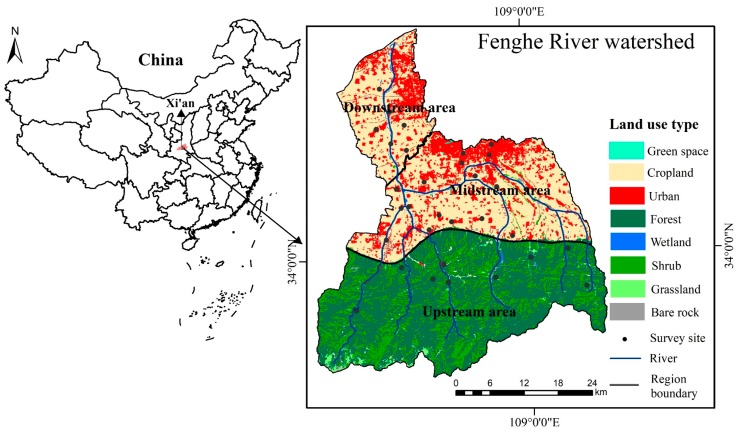
The location map of the Fenghe River watershed.

Fenghe River watershed is located in Xi’an City, Shaanxi Province, between 33°50′–34°24′N and 103°00′–105°30′E, with a total area of about 1708 km^2^ (Figure 1). We have added the downstream area to the area closely related to rivers, which is larger than the actual area of 1460 km^2^ [45]. There is a semi-humid climate in the region with an average annual temperature of about 15 °C and an average annual precipitation of about 800 mm, mostly concentrated in June to September. The elevation of the watershed is higher in the south and lower in the north. On the basis of the characteristics of the river and the landforms, ‘Round hill road’ divides the watershed into mountains and plains, and ‘Qinduzhen’ is the intersection of all tributaries. So, the watershed is divided into three regions: the upstream area, midstream area, and downstream area by the boundary of ‘Round hill road’ and ‘Qinduzhen’. The upstream area belongs to Qinling Mountains. It has various animal and plant types, rich BD, extremely important ecosystem functions, and abundant recreational resources. The midstream area is the transition area from mountain to plain, the land use is mainly for farmlands and towns. In recent years, urbanization has expanded rapidly, and some farmlands have been converted to urban lands. The downstream area belongs to Xi-Xi’an District. New recreational areas have been built, such as Kunming Pool, Shijingli characteristic town and Fenghe wetland park. They are close to the main urban areas of Xi’an, and attract a large number of tourists with convenient transportation. From upstream to downstream of the watershed, there are great differences in land use patterns and obvious changes in the ecological environment. It is a typical area that for studies on whether residential factors have an impact on residents’ perceptions for ES.

### 2.2. Sampling

In accordance with the existing literature on the setting of sample points and the actual situation of the watershed [46,47], we set the sample points. The upstream area belongs to the mountainous area, and there is less exchange between residents. Therefore, a village is randomly selected as a sample point in each valley, and sample points are appropriately added in densely populated river valleys. In the midstream and upstream areas, according to different socio-economic conditions, villages, city squares, and universities were randomly selected as sample points. According to the first law of geography, similar things are more closely related [48]. When we select sample points, we have a certain distance between the sample points in the space. This method of selecting sample points allows the sample to reflect the residents’ perceptions for ES as accurately as possible. We have set up 32 survey points, of which nine belong to the upstream area, 18 belongs to the midstream area, and five belong to the downstream area. The specific distribution is shown in Figure 1. According to the principle of minimum sample size selection, the following formula is used to calculate the minimum sample size [49]:

*N* = *Z*^2^ × (*P* × (1 − *P*))/*E*^2^ (1)

where *N* is the sample size; *Z* is the statistic; the confidence = 95%, *Z* = 1.96; the confidence = 90%; *Z* = 1.64; *E* is the error rate; and *P* is the probability value. On the basis of the error values of our previous 30 questionnaires, we used *Z* = 1.96, *E* = 5%, *P* = 0.5, then *N* = 384. The minimum sample size required for this study is 384.

### 2.3. Questionnaire Survey

According to the natural and social conditions of the Fenghe River watershed, this study selected BD, provisioning services (agriculture (AGR), food from livestock (LIV), fresh water (FW)), regulating services (water retention (WR), air purification (AP), soil retention (SR), water purification (WP)) and cultural services (aesthetics (AES), recreation (RE), spirit (SP)) as research subjects. A questionnaire was designed to study residents’ preferences and willingness to pay for ES and BD. From June to September 2018, we randomly distributed questionnaires to adult local residents (age ≥ 18 old) in 32 sample points. We simply described the purpose of the survey and the content of the questionnaire to these respondents, and then let them fill the questionnaire. For some older respondents who had difficulty in reading the questionnaire, we took the form of a question-and-answer session, while others adopted the form of self-completion. We obtained 386 valid questionnaires to meet the minimum sample size of 384.

The questionnaire was designed with reference to the questionnaires of Sherrouse et al. [23] and Castillo-Eguskitza et al. [26]. The questionnaire is divided into four parts: (1) residential factors: residential area, habitation, and residential time; (2) the residents’ preferences for ES and BD. First, we let the respondents select the five most important ES or BD for their own well-being. Then, we asked these respondents to rank ES which they chosen from 1 to 5, and 1 refers to the most important; (3) in order to further understand residents’ attitudes towards BD, regulating services and cultural services, we removed provisioning services and asked respondents whether they would be willing to pay for improving these ES or BD. If they agreed to pay, let them allocate 100 CNY (Chinese Yuan) to ES or BD (self-allocation, allowing allocation to one or more ES). The purpose of this design is to clearly understand the residents’ attitudes towards ES or BD. If they refused to pay, we asked them to provide the reasons for this; (4) the demographic characteristics of residents, such as gender, age, occupation, personal monthly income and education level. 

### 2.4. Data Processing and Analysis

Data processing is divided into four parts. First, the information of questionnaires was input into Excel 2016, and the basic information of residents was counted by SPSS Statistics 22. Second, the weighted scoring method was used to assess residents’ preferences for ES. ES selected for 1–5 was assigned 5–1 points, respectively, while unselected ES were assigned a score of 0, which were counted as *S_i_*. Each *S_i_* corresponded to a selection frequency *f_i_*, and ten ES and BD scores were obtained, i.e., importance (*I*). The greater the importance score, the higher the preference of residents for the ES. This calculation equation is as follows:

(2) $$I_{j}=\sum_{i=1}^{5}S_{i}\times f_{i}$$

where *I_j_* is the importance score of *j* ES; *i* is a sort of 1–5; *S_i_* is the score of *j* ES (1–5); *f_i_* is the frequency of selecting the *i*.

Third, the number of people who were willing and unwilling to pay for improving ES was counted. Since this survey method allowed the respondents to allocate a certain amount of money, the distribution itself had a weight. The total amount directly added was the amount that the residents were willing to pay for each ES, and the reasons for refusing to pay were also counted. The equation for calculating the amount of payment was as follows:

(3) $$PES_{j}=\sum_{i=1}^{n}X_{i}$$

where *PES_j_* is the amount willing to pay for *j* ES, *n* is the total number of people assigned to *j* ES; and *X_i_* is the amount allocated by the *i*-th respondent.

Fourth, the factors affecting preference heterogeneity were analyzed. Independent sample T-test was used to analyze the influence of gender and habitation. One-way ANOVA (F-test) was used to analyze the influence of age, personal monthly income, occupation, residential area, residential time, and education level. Spearman correlation analysis was used to analyze the influence of the ‘distance from the river ‘and the ‘distance from Xi’an city center’. The ‘distance from the river’ and ‘distance from Xi’an city center’ were obtained by ArcGIS 10.0 (ESRI, Redlands, CA, USA). 

## 3. Results

### 3.1. Basic Information of Respondents

The basic information of all respondents is shown in Table A1. This sample consisted of 178 women (46.1%) and 208 men (53.9%), roughly presenting the respondents’ gender balance. Regarding the age of respondents, the proportion of the age groups 18–30 years old accounted for the largest proportion (33.7%), while the proportion of other age groups (31–40, 41–50, 51–60, > 60 years old) were 16.3%, 18.4%, 15.8%, and 15.8%, respectively. On educational background, the interviewees had mainly received a middle school education (46.6%), followed by college and more than college education (32.4%). Respondents who had a primary school education alone accounted for 18.4%, and uneducated accounted for 2.6%. 

The income of all respondents varied significantly, only 5% respondents were paid more than 8000 CNY per month in 2018. The largest proportion (34.7%) of people had a monthly income of less than 2000 CNY, which was the lowest income group. About 24.1% and 25.1% of the sample earned a monthly income of 2000–3000 CNY and 3000–5000 CNY, while 11.1% had an income between 5000 and 8000 CNY every month. Regarding the occupation of interviewees, farmer accounted for the largest proportion (41.5%), followed by student (13.7%). Office clerk and self-employed had the same proportion (12.7%), meanwhile, services and public functionary also had a same proportion (5.7%). And retired and other occupation accounted for 4.1% and 3.6%, respectively.

Regarding the habitation status, most people (79.3%) lived in a rural area, and 20.7% lived in an urban area. Most people (72.5%) lived in the midstream area, and respondents living the upstream area and the downstream area accounted for 16.9% and 10.6%, respectively. In addition, a large proportion of respondents (29.8%) had lived for more than 50 years in the watershed, followed by between 21 and 30 years (26.7%). The residential time groups 31–40 and 41–50 were represented in similar proportions: 13.2% and 15%. About 9.6% and 5.7% of the sample lived in the area for 10–20 years and less than 10 years, respectively. 

### 3.2. Residents’ Preferences for ES

**Figure 2:**
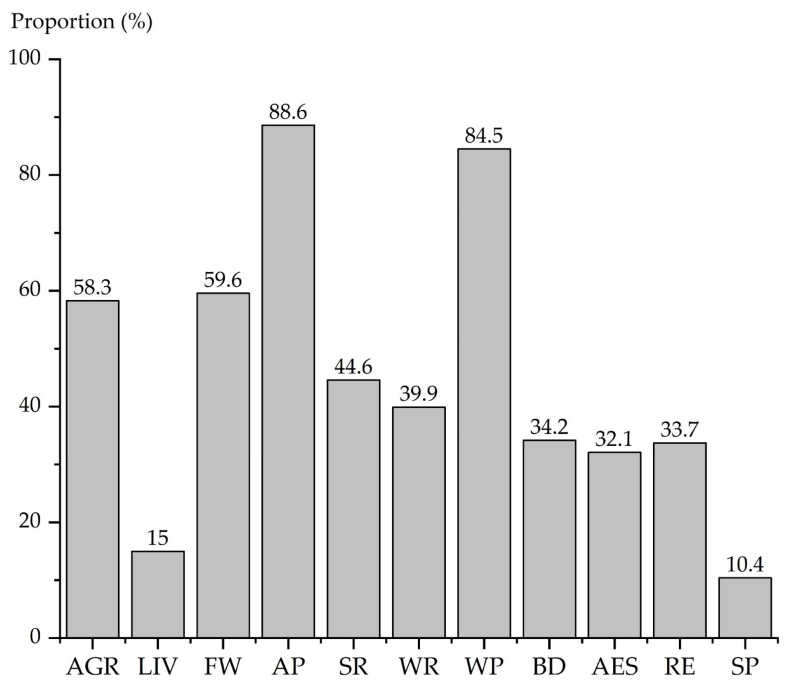
The proportion of respondents who chose ES and BD.

According to the respondents’ choices of ES and BD, the population distribution is shown in Figure 2. Regarding three provisioning services, FW and AGR were represented in roughly same proportions: 59.6% and 58.3%, respectively, while choosing LIV was only 15.0%. The proportion of four regulating services varied significantly. The proportion of residents choosing AP and WP was 88.6% and 84.5%, respectively, which was the highest proportion for two services. The proportion of residents choosing SR and WR accounted for 44.6% and 39.9%. In addition, there were few people choosing cultural services. The AES and RE had a similar proportion, 32.1% and 33.7%, respectively, and the SP had the lowest proportion (10.4%). Compared with ES, the proportion choosing BD were higher than that of cultural services, which was 33.7%.

**Figure 3:**
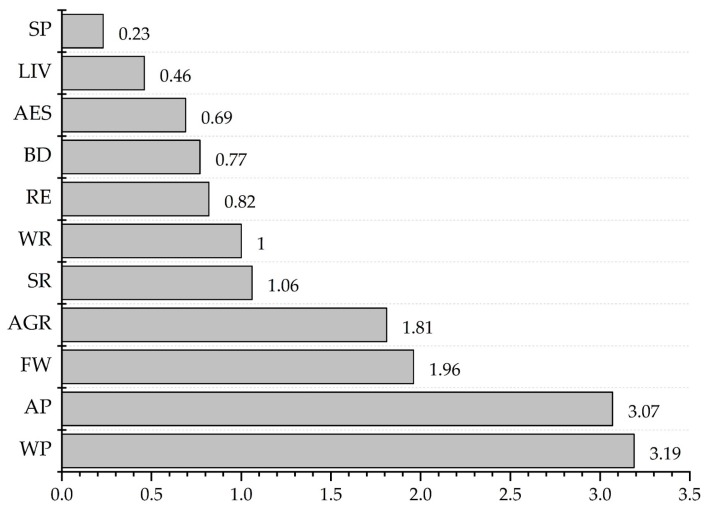
The importance scores of ten ES and BD.

Based on the equation of importance, the order and importance scores of ten ES and BD are displayed in Figure 3 and Table A2. The score of WP was 3.19, which was the highest, followed by AP (3.07). FW and AGR had a roughly same score: 1.96 and 1.81, respectively. Similarly, the scores of SR and WR were also roughly the same: 1.06 and 1.00, respectively. RE, AES and SP scored 0.82, 0.77, and 0.23, where SP had the lowest score among all of the options. BD and LIV scored 0.77 and 0.46.

### 3.3. Residents’ Willingness to Pay for Ecosystem Services

On the basis of the survey results, 198 respondents were willing to pay for improving ES, accounting for 51.3%. However, 188 respondents refused to pay, accounting for 48.7%. 

#### 3.3.1. Willingness to Pay

**Figure 4:**
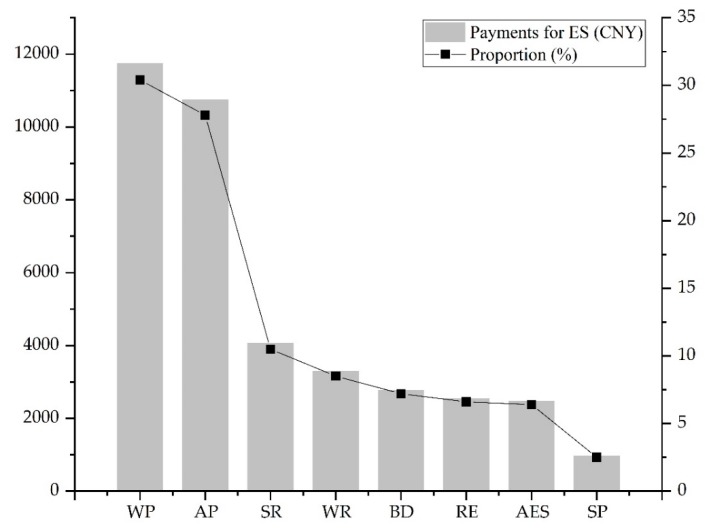
Total amount and number of people willing to pay for ecosystem services.

The amount and proportion of the willingness to pay of the residents is shown in Figure 4. The order of willingness to pay is: WP > AP > SR > WR > BD > RE > AES > SP. The amount allocation varied enormously. The amount of willing to pay was mainly concentrated on WP and AP. The amount of willing to pay for WP was about 12 times that of the SP. Moreover, the order of payment amount and importance was roughly the same.

#### 3.3.2. Reasons for Refusal to Pay

**Figure 5:**
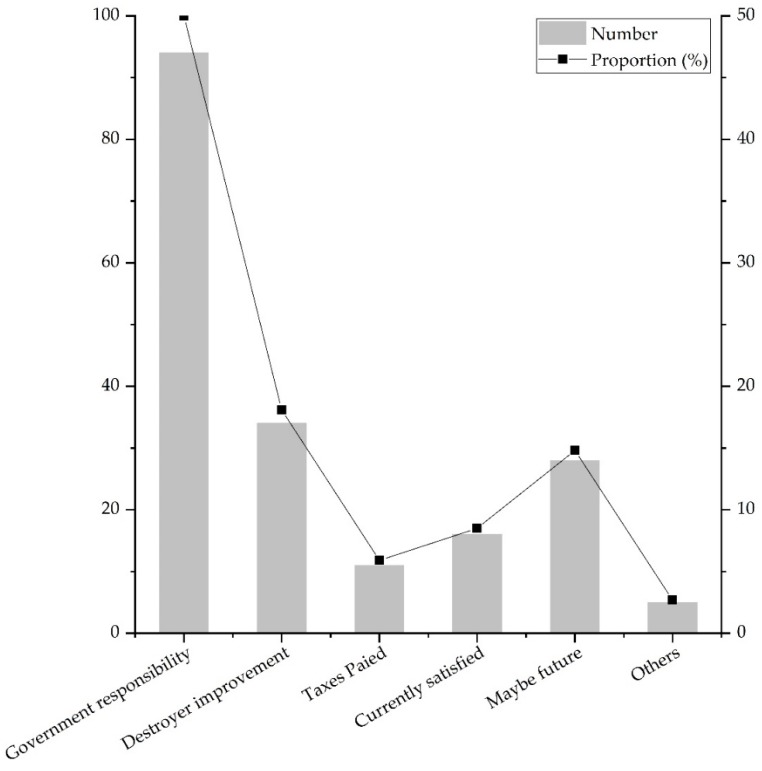
The quantity and proportion of different reasons for refusal to pay.

Based on the reasons that residents filled in, the reasons were divided into six categories, as shown in Figure 5. 94 people believed that improving ES was not the responsibility of the individual, but the government responsibility, accounting for 50% people who refused to pay. 34 people believed that the destroyers of ES should undertake the improvement of ES, i.e., destroyer improvement, accounting for 18.1%. 28 people said they were unwilling to pay at present and might pay in the future, accounting for 14.8%, of which 26 were students and said they were unable to afford to pay currently. 16 people were satisfied with the current ES and thought that there was no need for improvement, accounting for 8.5%. 11 people believed that personal tax had been paid, and the government should use a part of personal tax to improve ES, accounting for 5.9%. 5 people had another reason, accounting for 2.7%. 

### 3.4. Factors Affecting Preference Heterogeneity

#### 3.4.1. Personal Factors

The different levels of understanding for ES of residents caused differences in their preferences for ES, that is, preference heterogeneity (Table 1). The *p* values obtained showed that gender had no significant (*p* = 0.05) effect on all ES and BD. While, age had a significant (*p* = 0.001) influence on WR, WP and RE. Occupation had a significant (*p* = 0.001) impact on WR, WP, AES and BD, with a significant (*p* = 0.01) impact on RE. With the exceptions of LIV, FW, SR and SP, education level had a significant (*p* = 0.01) impact on all of the ES and BD factors. Personal monthly income had only a significant (*p* = 0.05) impact on FW, WP, BD, and RE.

To explore the specific effects of personal factors on preference heterogeneity, we calculated the grouping mean of each factor except gender (Table A3). For age factor, the preference for SR gradually increased with age, but the group of 51–60 years old became the lowest group. The older the age, the lower the preference for WR, the higher the preference for WP. The preference for RE roughly decreased with age, and the group of 31–40 years old was the group with the highest preference. Regarding education level, college and above groups had the lowest preference for AP, and other groups had less difference for AP. The higher the education level, the higher the preference for WR, but the lower the preference for WP. The preferences for AES and RE increased with the level of education. 

Residents of different occupations had different preferences for ES. Compared with other occupations, services and farmers had low preference for WR, but they had a higher preference for WP than other occupations. Public functionaries, students and retired persons had a high preference for BD, and other occupations had a lower preference for it. Public functionaries and students had a high preference for RE, and other occupations had a low preference; farmers had the lowest preference. As for income, the group with 2000–3000 CNY had a highest preference for FW and a lowest preference for BD. The group of less 2000 CNY had a highest preference for WP, and the group of 3001–5000 CNY and 5001–8000 CNY had a higher preference for RE.

#### 3.4.2. Residential Factors

We selected habitation, residential area, residential time, distance from the river, and distance from Xi’an City center as the residential factors. The analysis results are presented in Table 2. The ‘distance from the river’ had a significant impact on LIV, SR, WP (*p* = 0.01). The closer to the river, the higher preference for LIV, the lower preference for SR and WP. The ‘distance from Xi’an City center’ had a significant impact on LIV, SR, WP, and RE (*p* = 0.01). The closer to Xi’an City center, the lower the preference for LIV, and the higher the preference for SR and WP. Habitation had a significant impact on WP and RE (*p* = 0.001). Residential area had a significant impact on FW and RE (*p* = 0.001). In addition, the impact of residential time on WP and RE was very significant (*p* = 0.001).

Based on grouping mean of habitation, residential area and residential time, some information is shown in Table A4. Rural residents have a higher preference for WP than urban residents, and urban residents have a higher preference for RE than rural residents. The preference of upstream residents (0.92) for FW is significantly lower than that of midstream (2.25) and downstream (1.94) residents. Groups with a residential time of 21–30 years prefer WP much less than other groups. Groups with a residential time of 31–40 years have a higher preference for RE than other groups.

## 4. Discussion

### 4.1. Responders’ Reflections for the Perceptions of ES

Most respondents have higher responses to regulating services and provisioning services, such as AP, WP, AGR and FW, and relatively lower responses to cultural services and BD. According to the relevant literature [50], from 1960 to 2012, the number of haze days showed an increasing trend of fluctuation in Xi’an. In 2015, the number of “lightly above” pollution days reached 135 days. In 2016, the number of “lightly above” pollution days reached 206 days [51]. Therefore, the air quality in the Fenghe River watershed has threatened the safety of human survival, and residents have a higher preference for AP as a result. In addition, the water quality of the Fenghe River was seriously polluted from 2001 to 2006. Since 2006, the water quality was gradually improved. At present, the water quality has only reached the level 2 standard, meaning it is not suitable as drinking water [51]. Meanwhile, according to the “Water Quality Standard for Urban Water Supply” (CJ/T 206-2005), the Fe, Mn, NH_3_-N, pH, fluoride and total hardness in the source area of Fenghe River exceeded the standard [52]. So, residents also have a high preference for WP.

This result is inconsistent with Lhoest et al. [30], who found that local residents had a higher preference for provisioning services and cultural services in Cameroon, especially preference for meat. In our results, the residents of the Fenghe River watershed have a higher preference for AGR than LIV. This phenomenon shows that residents’ preferences for ES in different areas are influenced by local customs and dietary habits. He et al. [53] studied the ES preferences of Wuyi Mountain National Park and found that tourists have a higher preference for cultural services and a lower preference for other services. This difference is mainly because different stakeholders have different preferences for ES. From these differences, we can derive that people’s living areas and demands affect people’s perceptions for ES. Which is consistent with the study of de Juan et al. [54], who find that fishermen paid more attention to the destruction of coastal zones and habitat quality, while other residents and tourists paid more attention to the scenery of coastal zone. Kang et al. [55] also found that residents’ demand for ES had changed in different periods, and residents’ demand for cultural services had gradually increased with the development of the economy. These phenomena indicate that the respondents have different perceptions for ES due to different regional conditions, different stakeholders, and different periods. 

Based on Maslow’s hierarchy of needs [56], human needs are divided into five levels from low to high: physiological needs, safety and security needs, social needs, esteem needs and self-actualization needs. ES can provide three dimensions needs to human: physiological needs, safety and security needs, and self-actualization needs [57]. WP and AP are safety and security needs. Food and FW are physiological needs. RE, AES and SP are spirit needs. This study shows that as the economic level increases, people’s preferences for ES are shifting from physiological needs to safety needs and spirit needs. Therefore, the government should balance the needs of ES and seek regional sustainable development according to the local economic situation and the needs of residents.

### 4.2. Preference Heterogeneity 

#### 4.2.1. The Influence of Residential Factors

There are also differences in residents’ perceptions for ES in the watershed, that is, preference heterogeneity. Urban residents have a higher preference for AP than rural residents. This is because the main air pollutants in Xi’an are SO_2_, NO_2_, and TSP, and their main sources are motor vehicle exhaust and industrial production exhaust [58], which are more serious in urban areas than in rural areas. Residents in the midstream area have a higher preference for water quality than upstream and downstream residents. This is because industrial, agricultural, and domestic pollution is the main cause of water quality deterioration, and water pollution is the most serious issue in the midstream area [50]. Therefore, residential factors have a noticeable impact on residents’ preferences for ES. 

#### 4.2.2. The Influence of Personal Factors

Some studies have shown that individual characteristics such as gender, age, occupation, income, and education level had impacts on preferences [59,60,61]. This study also proves this phenomenon. In this study, gender has no significant effect on preferences, which is different from the study by Yang et al. [62]. They believe that female and male usually derive different benefits from ES, therefore, their perceptions for ES also differ. This may be caused by different ES provided by different regions. They mainly study the perceptions for ES provided by forests or mangroves. Although there are forests in our study area, due to the local policy, the forests provide regulating services and cultural services in Fenghe River watershed, hardly providing fibers and timber. This is the reason why the study differs from other scholars’ research. Human cognition is the active reflection of the subject on the object, influenced by direct and indirect experience. People occupy different environments and positions, and have different knowledge structures and cognitive abilities, and they also have different understandings of the same identified objects [63]. Generally speaking, people with higher education level have a deeper understanding of WR and BD, and have a higher preference for WR and BD. However, this was not the case in this study. The survey results show that people who are older and longer residence time have a higher preference for WP, which is related to their direct experience. They have witnessed the process of regional environmental change and have a higher preference for WP. The direct experience and indirect experience obtained by different occupations are obviously different, which is also the reason why occupation has the greatest influence on preference heterogeneity. The research of Zhang et al. also shows that people’s direct experience and local environment play an important role in shaping people’s understanding of their environment [33]. Therefore, personal factors also have impact on preference heterogeneity. Above all, preference heterogeneity is the result of multiple factors.

### 4.3. The Lower Awareness for WR, SR, and BD by Residents

Most respondents have a lower preference for WR, SR and BD. WR generally refers to measures to conserve water resources, is closely related to water resources, and plays an important role in regional water cycle and water balance, contributing to the sustainable use of water resources [64]. It means that the water retention ability directly affects the fresh water supply ability. However, local residents prefer FW to WR, which indicates that local residents have a lower level of understanding of WR and do not understand its important role in ecosystem functions. SR refers to the ability of ecosystems to control soil erosion and to maintain sediment accumulation [65]. Soil erosion can cause soil loss, the soil fertility decline, grain production decline, and river sediment increase, and can also lead to transverse movement of soil organic carbon, thereby affecting the global carbon cycle [66,67]. However, few residents can realize the importance of SR.

BD is an ecological complex of organisms and their habitats, which covers three levels: genetic diversity, species diversity and ecosystem diversity. Mace et al. believe that BD has three major roles: (1) regulators of ecosystem processes (e.g., bee pollination); (2) ultimate ES (e.g., planted apples); (3) commodities with their own value (e.g., ornamental value of wild animals and plants) [68]. From the perspective of the relationship between BD and ES, BD can be combined with the concepts of ES at all levels. It can support the ecosystem processes and directly impact the provision of some ES, and may be valuable itself. Although BD is very important, residents’ preferences and willingness to pay for BD are low, which indicates that most residents have not realized the importance of BD and have a low level of understanding of BD. 

As can be seen, residents prefer direct and local ES, and their level of understanding of indirect ES is low. Most residents do not recognize the important role of indirect ES and BD. This is consistent with the findings of Zhang et al., who conclude that the importance of provisioning services is generally recognized, while local residents have relatively low awareness of the need for regulating and supporting services [33].

### 4.4. Limitations

In order to ensure the sample can reflect the characteristics of the population, when we select sample points, we take into account the natural and socio-economic differences to select sample points, and then randomly distribute questionnaires at sample points. Although this survey method cannot guarantee the principle of complete randomness of the questionnaire, it can obtain the heterogeneity of residents’ perceptions for ES under different natural and socio-economic backgrounds. Although the randomness of this method needs to be improved, it can fully reflect the overall characteristics of the method. In future research, we will more realistically reflect the local residents’ perceptions for ES by increasing the sample points and sample size.

### 4.5. Management Recommendations

Based on our results and discussions, we put forward three suggestions for watershed management.

(1) the air quality and water quality need to be improved urgently. We should actively implement the haze control action plan in Xi’an, which involves fully implementing “coal cleaning”, dismantling coal-fired boilers and coal-fired facilities, reducing the use of loose coal, and so on. Meanwhile, we should also carry out the ‘ecological restoration project of the Fenghe River’, and actively build and expand sewage treatment plants to ensure that sewage discharge meets the standards.
(2) managers should raise residents’ awareness of regulating services and BD. Managers should educate residents about their important role and understand which actions protect or impair these services. For example, they should help residents understand that forests have the function of WR and SR, and then they will gradually form an ecological awareness of protecting forests.
(3) establishing a multi-channel fund system with government financial resources as the main source will help to improve the local ES and BD. The improvement of public assets, the main capital investment should be obtained from government funds, supplemented by other channels.

## 5. Conclusions

Taking the Fenghe River watershed as an example, this study applies social survey method to obtain the data of residents’ preferences and willingness to pay for ES. Using the ranking method, Likert scale method and hypothetical market method, the residents’ preferences and willingness to pay for ES were obtained. The independent sample T test, one-way ANOVA and Spearman correlation analysis were used to analyze preference heterogeneity. The results show that:

(1) Residents have a higher preference for WP, AP, AGR and FW, but a lower preference for WR, SR, BD and cultural services.
(2) About half of the residents are willing to pay, and half of residents are unwilling to pay. The amount that residents are willing to pay is roughly the same as their preferences for paying for services.
(3) Residents’ preferences for ES are heterogeneous, and residential factors and personal factors have an impact on heterogeneity.
(4) There are differences in residents’ perceptions for ES in different regions.
(5) Improving ES of the Fenghe River watershed should be carried out in three aspects: pollution control, raising residents’ awareness of ES, and funding guarantees.

Therefore, when making regional policies, it is essential to clearly understand the residents’ perceptions for ES, which will be beneficial for the sustainable use of ES.

## Figures and Tables

**Table 1:** *p* value and significance of variance analysis.

Factor	AGR	LIV	FW	AP	SR	WR	WP	BD	AES	RE	SP
Gender	0.577	0.448	0.206	0.945	0.041	0.842	0.628	0.276	0.960	0.756	0.541
Age	0.557	0.023 *	0.074	0.073	0.027 *	0.004 **	0.000 ***	0.163	0.153	0.000 ***	0.839
Occupation	0.104	0.095	0.060	0.511	0.140	0.000 ***	0.000 ***	0.000 ***	0.001 ***	0.008 **	0.260
Education	0.000 ***	0.364	0.173	0.001 ***	0.058	0.000 ***	0.000 ***	0.000 ***	0.007 **	0.000 ***	0.648
M-income	0.455	0.367	0.030 *	0.294	0.746	0.493	0.012 *	0.048 *	0.197	0.015 *	0.661

Note: * indicates a significant level of confidence at 0.05, ** indicates a significant level of confidence at 0.01, and *** indicates a significant level of confidence at 0.001; M-income means monthly income.

**Table 2:** Correlation coefficient, *p* value, and significance of residential factors.

Factor	Variable	AGR	LIV	FW	AP	SR	WR	WP	BD	AES	RE	SP
Habitation	P	0.319	0.108	0.049 *	0.156	0.262	0.051	0.000 ***	0.024 *	0.016 *	0.001 ***	0.535
RA	P	0.388	0.515	0.000 ***	0.213	0.222	0.146	0.146	0.157	0.05 *	0.000 ***	0.228
RT	P	0.778	0.180	0.01 *	0.036 *	0.201	0.018 *	0.000 ***	0.06	0.231	0.000 ***	0.473
DR	R	0.010	0.191 **	−0.062	−0.069	−0.175 **	0.122 *	−0.297 **	0.041	0.108 *	0.109 *	0.125 *
DXC	R	0.046	−0.190 **	0.049	0.069	0.145 **	−0.080	0.322 **	−0.090	−0.092	−0.160 **	−0.116 *

Note: RA = Residential area, RT = Residential time, DR = Distance from the river, Distance from Xi’an center, * indicates a significant level of confidence at 0.05, ** indicates a significant level of confidence at 0.01, and *** indicates a significant level of confidence at 0.001.

## Appendix A

**Table A1:** Basic information of respondents.

Item	Group	Number	Proportion (%)	Item	Group	Number	Proportion (%)
Gender	Male	208	53.9%	Education	Uneducated	10	2.6%
	Female	178	46.1%		Primary School	71	18.4%
Age	18–30	130	33.7%		Middle School	180	46.6%
	31–40	63	16.3%		≥College	125	32.4%
	41–50	71	18.4%	Residential time	<10 years	22	5.7%
	51–60	61	15.8%		10–20 years	37	9.6%
	>60	61	15.8%		21–30 years	103	26.7%
Monthly Income	<2000	134	34.7%		31–40 years	51	13.2%
	2000–3000	93	24.1%		41–50 years	58	15%
	3000–5000	97	25.1%		>50 years	115	29.8%
	5000–8000	43	11.1%	Occupation	Office Clerk	49	12.7%
	>8000	19	5%		Services	22	5.7%
Residential area	Upstream	65	16.9%		Public functionary	22	5.7%
	Midstream	280	72.5%		Self-employed	49	12.7%
	Downstream	41	10.6%		Student	53	13.7%
Habitation	Urban area	80	20.7%		Famer	160	41.5%
	Rural area	306	79.3%		Retired	16	4.1%
-	-	-	-		Other	14	3.6%

**Table A2:** The scores and order of ecosystem services.

ES	Selection Frequency						Importance	Order
	1	2	3	4	5	No Choice		
WP **	0.33	0.23	0.14	0.08	0.07	0.16	3.19	1
AP **	0.25	0.22	0.20	0.13	0.09	0.11	3.07	2
FW *	0.10	0.21	0.13	0.09	0.07	0.40	1.96	3
AGR *	0.15	0.09	0.12	0.12	0.10	0.42	1.81	4
SR **	0.02	0.06	0.11	0.12	0.13	0.55	1.06	5
WR **	0.03	0.07	0.09	0.09	0.12	0.60	1.00	6
RE ***	0.04	0.03	0.06	0.09	0.11	0.66	0.82	7
BD	0.02	0.02	0.07	0.12	0.11	0.66	0.77	8
AES ***	0.02	0.03	0.04	0.10	0.13	0.68	0.69	9
LIV *	0.02	0.04	0.03	0.05	0.01	0.85	0.46	10
SP ***	0.01	0.01	0.01	0.01	0.05	0.90	0.23	11

Note: * for the supply service, ** for the regulation service, *** for the cultural service.

**Table A3:** Mean value of each group of personal factors.

Factor	Sub-Factor	Number	AGR	LIV	FW	AP	SR	WR	WP	BD	AES	RE	SP
Education	No	10	2.60	0.40	2.9	3.10	1.7	0.20	4.1	0.30	0.20	0.00	0.30
	Primary school	71	2.38	0.23	2.34	3.13	1.46	0.62	3.83	0.42	0.44	0.32	0.21
	Middle school	180	1.66	0.51	1.98	3.46	0.94	1.03	3.43	0.59	0.68	0.79	0.18
	≥College	125	1.27	0.49	1.95	2.58	1.11	1.43	2.52	1.26	0.92	1.2	0.30
Age	18–30	130	1.51	0.67	1.86	2.94	1	1.34	2.62	0.98	0.88	1.08	0.28
	31–40	63	1.67	0.54	2.16	2.83	1	1.38	3.21	0.71	0.63	1.27	0.16
	41–50	71	1.70	0.21	1.77	3.54	1.30	0.85	3.61	0.68	0.76	0.73	0.21
	51–60	61	1.84	0.41	2.21	3.18	0.8	0.8	3.57	0.54	0.61	0.51	0.26
	>60	61	1.95	0.18	2.57	3.18	1.56	0.66	3.75	0.69	0.41	0.18	0.16
Monthly income	<2000	134	1..73	0.30	2.19	3.22	1.13	0.99	3.48	0.80	0.61	0.60	0.23
	2000–3000	93	1.82	0.44	2.44	3.08	1.26	0.91	3.29	0.44	0.58	0.62	0.16
	3001–5000	97	1.51	0.58	1.63	2.89	0.98	1.27	3.22	0.98	0.85	1.13	0.22
	5001–8000	43	1.49	0.51	1.81	3.05	1.07	1.19	2.37	0.91	1.00	1.28	0.26
	>8000	19	2.21	0.68	2.05	3.68	1.00	0.95	3.16	0.74	0.47	0.58	0.47
Occupation	Office clerk	49	1.63	0.94	1.88	2.82	1.29	1.47	3.08	0.76	0.49	1.06	0.29
	Services	22	1.55	0.32	1.64	3.55	1.45	0.73	3.55	0.73	0.65	0.73	0.23
	P-functionary	22	1.05	0.27	1.73	3.05	0.86	1.36	2.50	1.68	0.83	1.27	0.14
	Self-employed	49	1.86	0.53	1.71	3.33	1.04	1.53	2.76	0.59	0.58	0.82	0.37
	Student	53	1.19	0.36	2.09	2.85	0.74	1.49	2.57	1.25	1.09	1.30	0.23
	Farmer	160	1.97	0.32	2.43	3.19	1.27	0.61	3.86	0.51	0.42	0.53	0.14
	Retired	16	1.38	0.56	1.38	2.88	0.94	1.50	2.31	1.31	1.44	0.50	0.13
	Other	14	1.57	0.50	1.57	3.21	0.50	1.21	2.36	0.57	1.16	1.21	0.71

Note: P-functionary means public functionary.

**Table A4:** Mean value of each group of residential factors.

Factor	Sub-Factor	Number	AGR	LIV	FW	AP	SR	WR	WP	BD	AES	RE	SP
Habitation	Urban area	80	1.51	0.65	1.7	2.85	0.95	1.40	2.39	1.11	1.04	1.34	0.18
	Rural area	306	1.74	0.39	2.16	3.17	1.15	0.97	3.45	0.68	0.61	0.68	0.24
Residential Time	≤10 years	48	2.09	0.00	1.56	2.85	0.73	1.23	3.19	0.94	1.00	1.38	0.42
	11–20 years	41	1.30	0.30	1.63	3.20	1.10	1.20	3.15	1.02	0.71	1.07	0.24
	21–30 years	31	1.73	0.68	2.09	3.06	1.00	1.37	2.53	0.98	0.77	0.86	0.25
	31–40 years	49	1.51	0.61	2.18	2.67	1.10	1.35	3.02	0.82	0.84	1.31	0.10
	41–50 years	56	1.69	0.51	1.73	3.68	1.45	0.82	3.63	0.61	0.63	0.64	0.25
	>50 years	111	1.79	0.32	2.53	3.12	1.20	0.71	3.68	0.50	0.50	0.32	0.16
Residential Area	Upstream	67	1.57	0.30	0.92	3.06	1.03	1.39	1.39	0.67	0.70	0.84	0.22
	Midstream	280	1.76	0.47	2.25	3.06	1.18	1.00	1.00	0.74	0.64	0.66	0.20
	Downstream	39	1.38	0.51	1.94	3.54	0.77	0.95	0.95	1.13	1.15	1.92	0.44

## References

[1] Daily G.C. (1997). Nature’s Services: Societal dependence on natural ecosystems. Pac. Conserv. Biol..

[2] Fisher B., Turner R.K., Morling P. (2009). Defining and classifying ecosystem services for decision making. Ecol. Econ..

[3] Ellis E.C., Ramankutty N. (2008). Putting people in the map: Anthropogenic biomes of the world. Front. Ecol. Environ..

[4] Gao X., Shen J.Q., He W.J., Sun F.H., Zhang Z.F., Zhang X., Zhang C.C., Kong Y., An M., Yuan L. (2019). Changes in ecosystem services value and establishment of watershed ecological compensation standards. Int. J. Environ. Res. Public Health.

[5] Liu W., Zhan J., Zhao F., Yan H., Zhang F., Wei X. (2019). Impacts of urbanization-induced land-use changes on ecosystem services: A case study of the pearl river delta metropolitan region, China. Ecol. Indic..

[6] Costanza R., de Groot R., Sutton P., van der Ploeg S., Anderson S.J., Kubiszewski I., Farber S., Turner R.K. (2014). Changes in the global value of ecosystem services. Glob. Environ. Chang..

[7] Millennium Ecosystem Assessment (MEA) (2005). Ecosystems and Well-Being.

[8] Yang K., Yu Z., Luo Y., Zhou X., Shang C. (2019). Spatial-Temporal Variation of Lake Surface Water Temperature and its Driving Factors in Yunnan-Guizhou Plateau. Water Resour. Res..

[9] Knight A.T., Cowling R.M., Rouget M., Balmford A., Lombard A.T., Campbell B.M. (2008). Knowing but not doing: Selecting priority conservation areas and the research implementation gap. Conserv. Biol..

[10] McDonald R.I., Boucher T.M. (2011). Global development and the future of the protected area strategy. Biol. Conserv..

[11] Leverington F., Costa K.L., Pavese H., Lisle A., Hockings M. (2010). A global analysis of protected area management effectiveness. Environ. Manag..

[12] Willock J., Deary I.J., Edwards-Jones G., Gibson G.J., McGregor M.J., Sutherland A., Dent J.B., Morgan O., Grieve R. (1999). The role of attitudes and objectives in farmer decision making: Business and environmentally oriented behavior in Scotland. J. Agric. Econ..

[13] Reyers B., Roux D.J., Cowling R.M., Ginsburg A.E., Nel J.L., Farrell P.O. (2010). Conservation planning as a transdisciplinary process. Conserv. Biol..

[14] Mora C., Sale P.F. (2011). Ongoing global BD loss and the need to move beyond protected areas: A review of the technical and practical shortcomings of protected areas on land and sea. Mar. Ecol. Prog. Ser..

[15] Young J.C., Jordan A., Searle K.R., Butler A., Chapman D.S., Simmons P., Watt A.D. (2013). Does stakeholder involvement really benefit BD conservation?. Biol. Conserv..

[16] Reed M.S. (2008). Stakeholder participation for environmental management: Aliterature review. Biol. Conserv..

[17] Thompson B.S., Bladon A.J., Fahad Z.H., Mohsanin S., Koldewey H.J. (2016). Evaluation of the ecological effectiveness and social appropriateness of fishing regulations in the Bangladesh Sundarbans using a new multi-disciplinary assessment framework. Fish. Res..

[18] Harrison J.S., Bosse D.A., Phillips R.A. (2010). Managing for stakeholders, stakeholder utility functions, and competitive advantage. Strateg. Manag. J..

[19] De Vente J., Reed M.S., Stringer L., Valente S., Newig J. (2016). How does the context and design of participatory decision-making processes affect their outcomes? Evidence from sustainable land management in global drylands. Ecol. Soc..

[20] Asah S.T., Guerry A.D., Blahna D.J., Lawler J.J. (2014). Perception, acquisition and use of ecosystem services: Human behavior, and ecosystem management and policy implications. Ecosyst. Serv..

[21] Ciftcioglu G.C. (2017). Social preference-based valuation of the links between home gardens, ecosystem services, and human well-being in Lefke Region of North Cyprus. Ecosyst. Serv..

[22] Thompson B.S., Friess D.A. (2019). Stakeholder preferences for payments for ecosystem services (PES) versus other environmental management approaches for mangrove forests. J. Environ. Manag..

[23] Sherrouse B.C., Semmens D.J., Clement J.M. (2014). An application of Social Values for Ecosystem Services (SolVES) to three national forests in Colorado and Wyoming. Ecol. Indic..

[24] Zhang H., Gao Y., Hua Y., Zhang Y., Liu K. (2019). Assessing and mapping recreationists’ perceived social values for ecosystem services in the Qinling Mountains, China. Ecosyst. Serv..

[25] Burkhard B., Kroll F., Nedkov S., Müller F. (2012). Mapping ecosystem service supply, demand and budgets. Ecol. Indic..

[26] Castillo-Eguskitza N., Martín-López B., Onaindia M. (2018). A comprehensive assessment of ecosystem services: Integrating supply, demand and interest in the Urdaibai Biosphere Reserve. Ecol. Indic..

[27] Costanza R. (2000). Social goals and the valuation of ecosystem services. Ecosystems.

[28] Lamarque P., Tappeiner U., Turner C., Steinbacher M., Bardgett R.D., Szukics U., Schermer M., Lavorel S. (2011). Stakeholder perceptions of grassland ecosystem services in relation to knowledge on soil fertility and BD. Reg. Environ. Chang..

[29] Martín-López B., Iniesta-Arandia I., García-Llorente M., Palomo I., Casado-Arzuaga I., Amo D.G.D., Gómez-Baggethun E., Oteros-Rozas E., Palacios-Agundez I., Willaarts B. (2012). Uncovering ecosystem service bundles through social preferences. PLoS ONE.

[30] Muhamad D., Okubo S., Harashina K., Parikesit Gunawan B., Takeuchi K. (2014). Living close to forests enhances people’s perception of ecosystem services in a forest–agricultural landscape of West Java, Indonesia. Ecosyst. Serv..

[31] Lhoest S., Dufrêne M., Vermeulen C., Oszwald J., Doucet J., Fayolle A. (2019). Perceptions of ecosystem services provided by tropical forests to local populations in Cameroon. Ecosyst. Serv..

[32] Geijzendorffer I.R., Roche P.K. (2014). The relevant scales of ecosystem services demand. Ecosyst. Serv..

[33] Zhang W., Kato E., Bhandry P., Nkonya E., Ibrahim H.I., Agbonlahor M., Ibrahim H.Y., Cox C. (2016). Awareness and perceptions of ecosystem services in relation to land use types: Evidence from rural communities in Nigeria. Ecosyst. Serv..

[34] De Groot R.S., Wilson M.A., Boumans R.M. (2002). A typology for the classification, description and valuation of ecosystem functions, goods and services. Ecol. Econ..

[35] Wilson M.A., Carpenter S.R. (1999). Economic valuation of freshwater ecosystem services in the United States: 1971–1997. Ecol. Appl..

[36] Kremen C., Ostfeld R.S. (2005). A call to ecologists: Measuring, analyzing, and managing ecosystem services. Front. Ecol. Environ..

[37] Boeraeve F., Dendoncker N., Sander J., Gómez-Baggethun E., Dufrêne M. (2015). How (not) to perform ecosystem service valuations: Pricing gorillas in the mist. Biodivers. Conserv..

[38] Orenstein D.E., Groner E. (2014). In the eye of the stakeholder: Changes in perceptions of ecosystem services across an international border. Ecosyst. Serv..

[39] Braat L.C., de Groot R. (2012). The ecosystem services agenda: Bridging the worlds of natural science and economics, conservation and development, and public and private policy. Ecosyst. Serv..

[40] Collins S.L., Carpenter S.R., Swinton S.M., Orenstein D.E., Childers D.L., Gragson T.L., Grimm N.B., Grove J.M., Harlan S.L., Kaye J.P. (2010). An integrated conceptual framework for long-term social-ecological research. Front. Ecol. Environ..

[41] Hartter J., Solomon J., Ryan S.J., Jacobson S.K., Goldman A. (2014). Contrasting perceptions of ecosystem services of an African forest park. Environ. Conserv..

[42] Iftekhar M.S., Takama T. (2007). Perceptions of BD, environmental services, and conservation of planted mangroves: A case study on Nijhum Dwip Island. Bangladesh. Wetl. Ecol. Manag..

[43] Lu S.D., Sun Y.J., Zhao X., Wang L., Ding A.Z., Zhao X.H. (2016). Sequencing insights into microbial communities in the water and sediments of Fenghe River, China. Arch. Environ. Contam. Toxicol..

[44] Bhandari P., Kc M., Shrestha S., Aryal A., Shrestha U.B. (2016). Assessments of ecosystem service indicators and stakeholder’s willingness to pay for selected ecosystem services in the Chure region of Nepal. Appl. Geogr..

[45] Zhang F.P., Zhao S., Zhou Z.C., Wei Y.F. (2014). Relationship between changes of land use pattern and water quality in Fenghe River watershed. Bull. Soil Water Conserv..

[46] Belton B., Filipski M. (2019). Rural transformation in central Myanmar: By how much, and for whom?. J. Rural Stud..

[47] Wei H.J., Liu H.M., Xu Z.H., Ren J.H., Lu N.C., Fan W.G., Zhang P., Dong X.B. (2018). Linking ecosystem services supply, social demand and human well-being in a typical mountain–oasis–desert area, Xinjiang, China. Ecosyst. Serv..

[48] Tobler W.R. (1970). A Computer Movie Simulating Urban Growth in the Detroit Region. Econ. Geogr..

[49] Walpole R.E., Myers R.H. (1978). Probability and Statistics for Engineers and Scientists.

[50] Wang S., Xiu T.Y., Sun Y., Meng X.R., Xu J.C. (2014). The changes of mist and haze days and meteorological element during 1960–2012 in Xi’an. Acta Sci. Circumstantiae.

[51] Li Y.J., Guo W.J., Dong W., Li J.K. (2014). Water environmental characteristics and pollution constitution for the Fenghe river in Shaanxi province. China Rural Water Hydropower.

[52] Meng D.F., Tong F., Wang X.Y. (2018). Application of entropy weight extended set pair analysis model on Xi’an city shallow groundwater quality assessment. Coal Geol. China.

[53] He S.Y., Su Y., Wang L., Cheng H.G. (2019). Realisation of recreation in national parks: Perspective of ecosystem services demand and willingness to pay of tourists in Wuyishan Pilot. J. Nat. Resour..

[54] De Juan S., Gelcich S., Fernandez M. (2017). Integrating stakeholder perceptions and preferences on ecosystem services in the management of coastal areas. Ocean Coast. Manag..

[55] Kang Y., Cheng C.X., Liu X.H., Zhang F., Li Z.H., Lu S.Q. (2019). An ecosystem services value assessment of land-use change in Chengdu: Based on a modification of scarcity factor. Phys. Chem. Earth.

[56] Maslow A.H. (1943). A Theory of Human Motivation. Psychol. Rev..

[57] Dominati E., Patterson M., Mackay A. (2010). A framework for classifying and quantifying the natural capital and ecosystem services of soils. Ecol. Econ..

[58] Li P.W. (2018). Study on Investment Performance Evaluation and Influencing Factors of Air Pollution Control in Xi’an. Master’s Thesis.

[59] Brun M., Di Pietro F., Bonthoux S. (2018). Residents’ perceptions and valuations of urban wastelands are influenced by vegetation structure. Urban For. Urban Green..

[60] Gobster P.H., Nassauer J.I., Daniel T.C., Fry G. (2007). The shared landscape: What does aesthetics have to do with ecology?. Landsc. Ecol..

[61] Stern P.C. (2000). Toward a coherent theory of environmentally significant behavior. J. Soc. Issues.

[62] Yang Y.C.E., Passarelli S., Lovell R.J., Ringler C. (2018). Gendered perspectives of ecosystem services: A systematic review. Ecosyst. Serv..

[63] Marx K., Engels F. (1995). Selected Works of Marx and Engels.

[64] Sun X., Lu Z.M., Li F., Crittenden J.C. (2018). Analyzing spatio-temporal changes and trade-offs to support the supply of multiple ecosystem services in Beijing, China. Ecol. Indic..

[65] Costanza R., D’Arge R., de Groot R., Farber S., Grasso M., Hannon B., Limburg K., Naeem S., O’Neill R.V., Paruelo J. (1997). The value of the world’s ecosystem services and natural capital. Nature.

[66] Van Oost K., Verstraeten G., Doetterl S., Notebaert B., Wiaux F., Broothaerts N., Six J. (2012). Legacy of human-induced C erosion and burial on soil-atmosphere C exchange. Proc. Natl. Acad. Sci. USA.

[67] Li P., Han Z., Jia X., Mei Z., Han X., Wang Z. (2019). Comparative analysis of an organic Rankine cycle with different turbine efficiency models based on multi-objective optimization. Energy Convers. Manag..

[68] Mace G.M., Norris K., Fitter A.H. (2012). BD and ecosystem services: A multilayered relationship. Trends Ecol. Evol..

